# Co-Directional Chromosomal Clustering of Metabolic Pathway Genes as a Layout Prior for Synthetic Construct Design

**DOI:** 10.3390/biotech15040075

**Published:** 2026-09-03

**Authors:** Xiaowei Han, Zhixu Qiu, Jiani Hu, Jie Song

**Affiliations:** 1 College of Life Sciences, Northwest A&F University, Yangling 712100, China; hanxiaowei@chd.edu.cn (X.H.);; 2 School of Chemistry and Molecular Biosciences, The University of Queensland, Brisbane, QLD 4072, Australia; jiani.hu@uqconnect.edu.au

**Keywords:** synteny, operon, metabolic pathway design, synthetic biology, gene cluster, BioCyc, gene order, layout prior, SynPAL

## Abstract

Metabolic pathway design tools largely address reaction routes and enzyme choice, whereas a complementary set of questions—how pathway genes are arranged on bacterial chromosomes and whether co-directional neighborhoods can inform multi-gene construct layout—has received comparatively little systematic treatment. Here, we present SynPAL (Synteny-Informed Pathway Assembly and Layout), a computational analysis that scores co-directional clustering of BioCyc pathway genes from genomic coordinates (same replicon and strand, intergenic distance ≤ 2000 bp) and translates the resulting scores into design-oriented hypotheses. On a core panel of 55 prokaryotes (887 analyzable pathways), medium or high clustering occurs for 290 pathways (32.7%), with a mean cluster score of 0.432 versus 0.262 under an organism-matched random-gene-set null; 692/887 pathways remain significant after Benjamini–Hochberg false-discovery-rate control at q<0.05. High scores recover classical operons, including *nan*, *bkd*, *pdxST*, and *gmd*–*fcl*, under a fixed labeling protocol. Scoring gene-name order in pathway tables instead of coordinates yields substantially higher medium/high rates on the same pathways (80.1% vs. 32.0% on the full 55-organism panel; 90.5% vs. 49.2% on a paired 63-pathway set) and correlates only weakly with coordinate scores (Pearson’s r=0.23), demonstrating that table order is not chromosomal synteny. Pathways are assigned design-readiness tiers T1–T4 as layout hypotheses, and sequence-backed construct drafts were generated for 222 of 224 T1 pathways. An expanded survey of 9071 prokaryotic databases (1629 pathways) shows a similar selective landscape (42.7% medium/high). SynPAL does not select enzymes, predict flux, or report wet-lab expression; it supplies coordinate-based cluster scores, tiers, and draft layouts that can accept a gene list from reaction-network CAD tools.

## 1. Introduction

Heterologous multi-gene pathways remain central to metabolic engineering, yet most computational design effort addresses reaction routes rather than gene arrangement. Retrosynthesis and pathway-search systems such as RetroPath and RetroPath2.0 [1,2], reinforcement-learning-based bioretrosynthesis [3], RetSynth [4], and the Galaxy-SynBioCAD portal [5] propose enzymes and, in some pipelines, DNA parts for chemical transformations. They primarily answer the question of which genes to use. Practitioners still decide how those genes should be situated relative to one another, often by consulting known operons or conducting ad hoc genomic inspection.

In bacteria, co-transcribed clusters (operons) are a primary organizational form of gene regulation [6,7]. Conserved neighborhoods predict functional coupling [8,9], even though global gene order is rearranged more readily than gene content [10]. Intergenic distance on the same strand is an informative operon signal [11,12]. Gene order within operons can match protein-complex assembly order [13], and experimental rearrangement of pathway genes alters expression balance and product titers [14,15,16]. Horizontal-transfer and co-regulation models treat compact clusters as evolutionary modules [17,18]. Synthetic biology continues to use operon architecture as a circuit abstraction [7], and recent polycistronic expression work underscores layout as a design variable [19].

These observations motivate a layout prior, not a validated portability theorem: gene sets that remain co-directional across species are reasonable starting architectures for multi-gene constructs. Implementing that prior requires chromosomal coordinates rather than database display order, statistical controls against chance co-localization under a stated null, a mapping from conservation to design options, and optional sequence drafts for experimental follow-up.

We introduce **SynPAL** (Synteny-Informed Pathway Assembly and Layout). SynPAL quantifies co-directional synteny of metabolic pathways on a core panel of 55 prokaryotic BioCyc pathway/genome databases [20,21], tests enrichment under an explicit gene-set-size null with false-discovery-rate control, compares high scores with those of documented operons under a fixed labeling protocol, assigns design-readiness tiers T1–T4 as layout hypotheses, and produces construct drafts for single-cassette (T1) pathways. An expanded prokaryotic survey checks the robustness of the conservation landscape. Open software supports re-scoring of user-defined gene sets on user-supplied genomes. SynPAL does not replace enzyme selection, thermodynamic ranking, flux analysis, or chassis-specific expression optimization.

## 2. Materials and Methods

### 2.1. Pathway and Genome Data

Pathway membership and gene coordinates were taken from BioCyc pathway/genome databases (PGDBs) [20,21,22]. Gene positions were read primarily from PGDB gene tables, with gene-object coordinate fields used when needed. Whole-replicon nucleotide sequences distributed with each PGDB supported optional sequence extraction for construct drafts.

The **core panel** comprises 55 prokaryotic PGDBs (52 from bacteria and 3 from archaea) selected for pathway annotation and genome availability; eukaryotic databases and MetaCyc were excluded from organism counts. Primary statistics, null models, design extraction, and the reference SQLite resource are based on this panel.

The **expanded panel** applies the same scoring procedure to prokaryotic PGDBs classifiable as bacteria or archaea in a BioCyc v26 snapshot (*n* = 9071 after domain filtering). It is used only for conservation scores and tier counts.

### 2.2. Co-Directional Clusters

For each organism and pathway, pathway genes were mapped to chromosomal intervals (one-based inclusive). A **co-directional cluster** is a maximal set of mapped genes on the same replicon and strand with successive intergenic gaps ≤ max_gap (default 2000 bp), following distance scales used in operon prediction [11,12]. Operon inference remains an active area of resource development in the BioCyc ecosystem [23]. Cross-genome gene matching uses shared gene symbols and therefore undercalls orthology when naming differs; scores are lower bounds on true neighborhood conservation.

### 2.3. Conservation Scores

For organism *i* with $m_{i}\geq 2$ mapped pathway genes and largest co-directional cluster size $c_{i}$, the organism-level ratio is $r_{i}=c_{i}/m_{i}$. The pathway-level

(1) $$\mathrm{cluster}\_\mathrm{score}={\mathrm{mean}}_{i}\left(r_{i}\right)$$

averages $r_{i}$ over organisms with $m_{i}\geq 2$. When comparable orders exist, order_score is Kendall’s coefficient of concordance for transcriptional order within clusters. We use

(2) synteny_score=0.6cluster_score+0.4order_score,

falling back to cluster_score alone when order cannot be scored. The levels of cluster_score are defined as HIGH (≥0.7), MEDIUM ([0.5, 0.7)), LOW ([0.3, 0.5)), and VERY_LOW (<0.3). A pathway is analyzable if at least two organisms each contribute at least two mapped genes.

### 2.4. Null Model and Multiple Testing

For each organism and gene-set size *k*, we drew random *k*-gene sets uniformly without replacement from that organism’s full gene inventory (200 draws), applied the same co-directional clustering rule, and recorded mean cluster ratios. Pathway-level *p*-values used 500 Monte Carlo averages that sample one random organism-level ratio per qualifying organism (add-one smoothing; seed 42).

This null holds constant organism identity and gene-set size *k*. It does not hold constant local gene density, strand composition of a genomic region, or other spatial structure beyond what random gene sampling implies. Enrichment relative to this null therefore indicates that pathway gene sets cluster more than random gene sets of the same size in the same genomes; it is not a complete model of sequence composition or regional density.

Pathway-level *p*-values were adjusted by the Benjamini–Hochberg false-discovery-rate (FDR) procedure. We report both unadjusted p<0.05 counts and FDR q<0.05 counts.

With 500 Monte Carlo averages and add-one smoothing, the smallest attainable *p*-value is approximately 0.002; 618 of 887 pathways (69.7%) sit at this resolution floor. The Benjamini–Hochberg procedure remains stable under such ties in this dataset: all 618 floor pathways fall within the rejection set at q<0.05 (692/887; 78.0%), and the rank at which the step-up threshold is crossed lies beyond the tied block. Because the organism-level random draws are reused across pathways of equal gene-set size *k*, the resulting pathway-level *p*-values are not strictly independent; the FDR figure should therefore be read as an empirical enrichment rate under the stated null rather than as a fully independent test. Local gene density, strand composition, and replicon architecture are not held constant by the size-only null, and so a portion of the observed signal could reflect regional gene-context effects in genomes with unusually dense or strand-biased neighborhoods.

### 2.5. Design-Readiness Tiers (Layout Hypotheses)

Let $R=\max_{i}r_{i}$ and $C=\max_{i}c_{i}$ over organisms with $m_{i}\geq 2$. Tiers summarize clustering patterns as **layout hypotheses** for construct design, not experimental portability classes:

- **T1**: *R* ≥ 0.999—at least one organism places the full mapped set in a single co-directional cluster (single-cassette layout candidate).
- **T4**: *C* < 2—no two-gene cluster is observed (native clustering provides little layout guidance).
- **T2**: neither T1 nor T4, and cluster_score ≥ 0.5—partial modules recur (multi-cassette, often same-host candidates).
- **T3**: neither T1 nor T4, and cluster_score < 0.5—clusters exist but are weakly conserved (multi-species module candidates).

### 2.6. Construct Drafts

Given a pathway and organism set, the design procedure enumerates co-directional clusters; ranks modules (completeness, size, annotation richness, reference-strain preference, and consensus-order agreement); optionally seeks a compact multi-module cover; extracts the sequence with neighbor-limited flanks clamped to [40, 800] bp; and writes FASTA, GFF3, and GenBank records with Gibson-oriented junction primers based on a Wallace melting-temperature heuristic [24]. The primers are structural drafts. The drafts are computational sequence records, not experimentally validated constructs. The Wallace rule does not model salt, hairpin, or primer-dimer effects, and amplification efficiency may be reduced for long Gibson junctions or GC-rich template regions; we recommend re-deriving oligos with Primer3 [25] or an equivalent tool before ordering.

### 2.7. Column-Order Control

As a methodological control, we scored “conservation” from the order of gene names in BioCyc pathway tables rather than from chromosomal coordinates. Two panels were analyzed to separate the effect of panel composition from the effect of the metric itself. First was the full 55-organism core panel: a direct re-run of the column-order metric on all 55 PGDBs yielded 682 analyzable pathways, with 70.5% MEDIUM/HIGH under legacy column-order thresholds (HIGH ≥ 0.9, MEDIUM ≥ 0.7) and 80.1% under thresholds aligned with the coordinate metric (HIGH ≥ 0.7, MEDIUM ≥ 0.5). On the same 682 pathways, the coordinate cluster score reached MEDIUM/HIGH in only 32.0%—a 48-percentage-point gap. Second, to match the 45-organism deduplicated species panel used in earlier work (one PGDB per species), we repeated the comparison on that subset (569 analyzable pathways; 72.4% medium/high column order under legacy thresholds vs. 29.7% coordinate on the same 569), confirming that panel composition does not change the qualitative conclusion. For a directly paired scatter comparison of the two metrics, we additionally report column-order and coordinate scores on the same 63 pathways that were analyzable in both the legacy pipeline and the earlier synteny scan (79.4% legacy/90.5% aligned vs. 49.2% coordinate on the same 63; Pearson’s r=0.23). The key observation is robust across all three configurations: gene-name column order yields substantially higher MEDIUM/HIGH rates than coordinate-defined clustering on the very same pathway sets, and so pathway-table order is not a proxy for chromosomal synteny.

### 2.8. Operon Concordance Protocol

For literature concordance, we labeled the dominant co-directional cluster of each HIGH pathway with $n_{\mathrm{organisms}}\geq 5$ (*n* = 47) as confirmed, partial, unknown, or contradicted by comparing it with independent literature or curated resources, using fixed label definitions stored with the analysis (operon-like co-transcription or multi-gene functional unit for the dominant cluster genes, not the entire BioCyc pathway name). A random VERY_LOW sample (initially *n* = 15, expanded to *n* = 30 by adding 15 additional random VERY_LOW pathways, seed 42) used the same labels. Labeling prioritizes honest *unknown* classification over forced confirmation. Support is defined as a *confirmed* or *partial* classification. One-sided Fisher’s exact tests compare support rates between the HIGH and VERY_LOW sets. This is a targeted concordance check, not an unbiased genome-wide discovery of novel operons. The control set was expanded from *n* = 15 to *n* = 30 in response to reviewer comments; support rates should still be interpreted as protocol-defined enrichment among high-scoring pathways rather than as discovery precision across the full pathway set.

### 2.9. Benchmark Against BioCyc Transcription Units

As an independent benchmark that covers all 887 pathways (not only HIGH subsets), we compared SynPAL’s largest co-directional cluster for each (pathway, organism) pair against the transcription unit (TU) annotations present in BioCyc PGDBs. For 52 of 55 PGDBs, genes.dat contains COMPONENT-OF lines linking genes to TU identifiers (255,598 gene–TU pairs across 187,618 genes). For each cluster with ≥2 genes and TU annotation available (*n* = 4906 of 6065 clusters), we asked whether ≥2 of the SynPAL cluster genes shared a common TU identifier, yielding a per-cluster binary agreement score. This metric is a precision measure: it asks whether SynPAL co-directional clusters correspond to BioCyc curations of transcriptional units.

### 2.10. Assembly Feasibility Check

Each of the 222 T1 construct FASTA files was parsed to extract (i) total nucleotide length, (ii) GC content, (iii) number of records, (iv) number and Tm of junction primers, and (v) overlap length between adjacent fragments. A construct passes the assembly feasibility check if it meets the following criteria: total length ≤ 30,000 bp (single-pot Gibson range [24]), GC content in [0.25, 0.75], at least one primer per fragment, Tm in [40, 80] °C, and minimum overlap ≥ 15 bp.

### 2.11. Scope, Hand-Off, and Software

SynPAL accepts a pathway gene list (from BioCyc or a user list) plus genome annotations and optional FASTA. It returns cluster scores, tiers, and optional sequence drafts. It does not perform enzyme selection, retrosynthesis, FBA, promoter prediction, or chassis fitness scoring. A typical hand-off is as follows: reaction-network CAD → gene symbols → SynPAL layout scores/drafts → experimental design tools.

Analyses are implemented in Python (version 3.10.8). A small open toolkit exposes scoring and draft generation for user GFF/GTF + FASTA; core-panel scores are stored in SQLite. A static website (https://synpal.sjtu.bio, accessed on 28 August 2026) provides browsing of derived scores and drafts. Sequence drafts are regenerated from licensed BioCyc installs or user genomes at analysis time; public redistribution follows derived-score and draft policies documented with the software release rather than bulk PGDB flat files.

## 3. Results

### 3.1. A Selective Conservation Landscape

**Figure 1:**
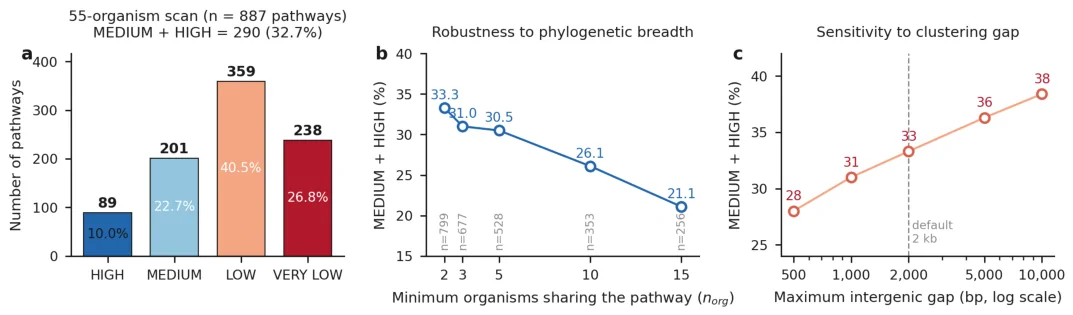
Pathway-level chromosomal clustering on the core panel. (**a**) Distribution of 887 analyzable pathways across conservation levels (levels follow the mean cluster score); (**b**) robustness of the MEDIUM + HIGH fraction to the minimum number of organisms sharing a pathway (45-organism panel); (**c**) sensitivity of the MEDIUM + HIGH fraction to the maximum intergenic gap (log scale; dashed line marks the 2 kb default; 45-organism panel). Medium and high together comprise 32.7% of 887 pathways.

The core analysis covered 55 prokaryotic PGDBs and 887 analyzable pathways (max_gap = 2000 bp). Of these, 89 (10.0%) were HIGH, 201 (22.7%) MEDIUM, 359 (40.5%) LOW, and 238 (26.8%) VERY_LOW; thus, 290 pathways (32.7%) fell in the MEDIUM or HIGH range (Figure 1, Table 1). Order conservation (order_score ≥ 0.7) occurred for 229 pathways. Organism coverage per pathway varied widely (median = 9; mean = 14.7). A substantial minority of pathways therefore carry a strong co-directional signal, while most remain partly or fully scattered.

### 3.2. Clustering Exceeds That of a Size-Matched Null

**Figure 2:**
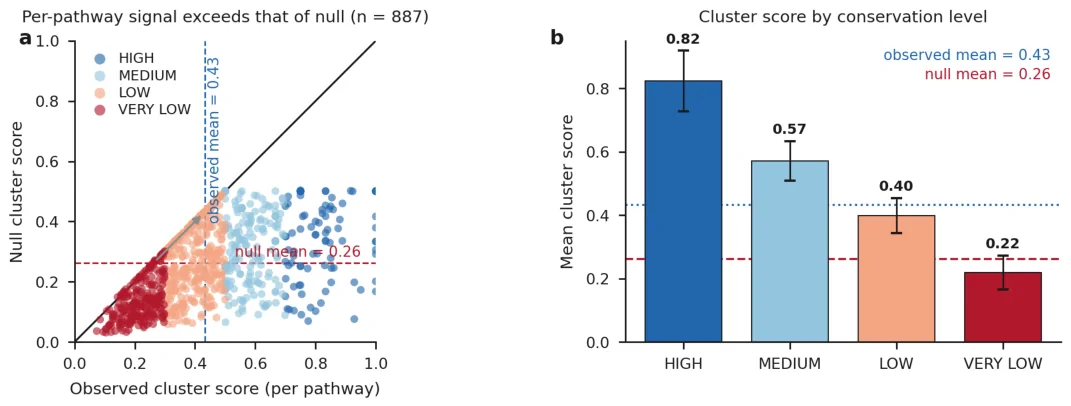
Observed cluster scores versus size-matched random gene sets within the same genomes. (**a**) Per-pathway observed versus null cluster score; points are colored by conservation level, the diagonal marks equality, and dashed lines mark the grand means. (**b**) Mean cluster score by conservation level (error bars: SD); dashed and dotted lines mark the null and observed grand means, respectively.

The mean observed cluster score was 0.432, compared with 0.262 under the organism- and size-matched random-gene-set null (difference 0.170). At unadjusted p<0.05, 697/887 pathways (78.6%) were significant, including 89/89 HIGH pathways. After Benjamini–Hochberg FDR control, 692/887 pathways (78.0%) remained significant at q<0.05; all 89 HIGH pathways retained q<0.05 (Figure 2). A sensitivity analysis with a density-matched null—random *k*-gene sets drawn from genomic windows of similar local gene density rather than from the full genome, run on a seven-organism subset (710 pathways)—confirmed that the observed cluster score (0.434) still substantially exceeds that of the density-matched null (0.241; gap 0.193), which is slightly higher than that of the original random null on the same subset (0.208), indicating that regional gene density alone cannot account for the observed co-directional clustering signal.

### 3.3. High Scores Recover Documented Operons

**Figure 3:**
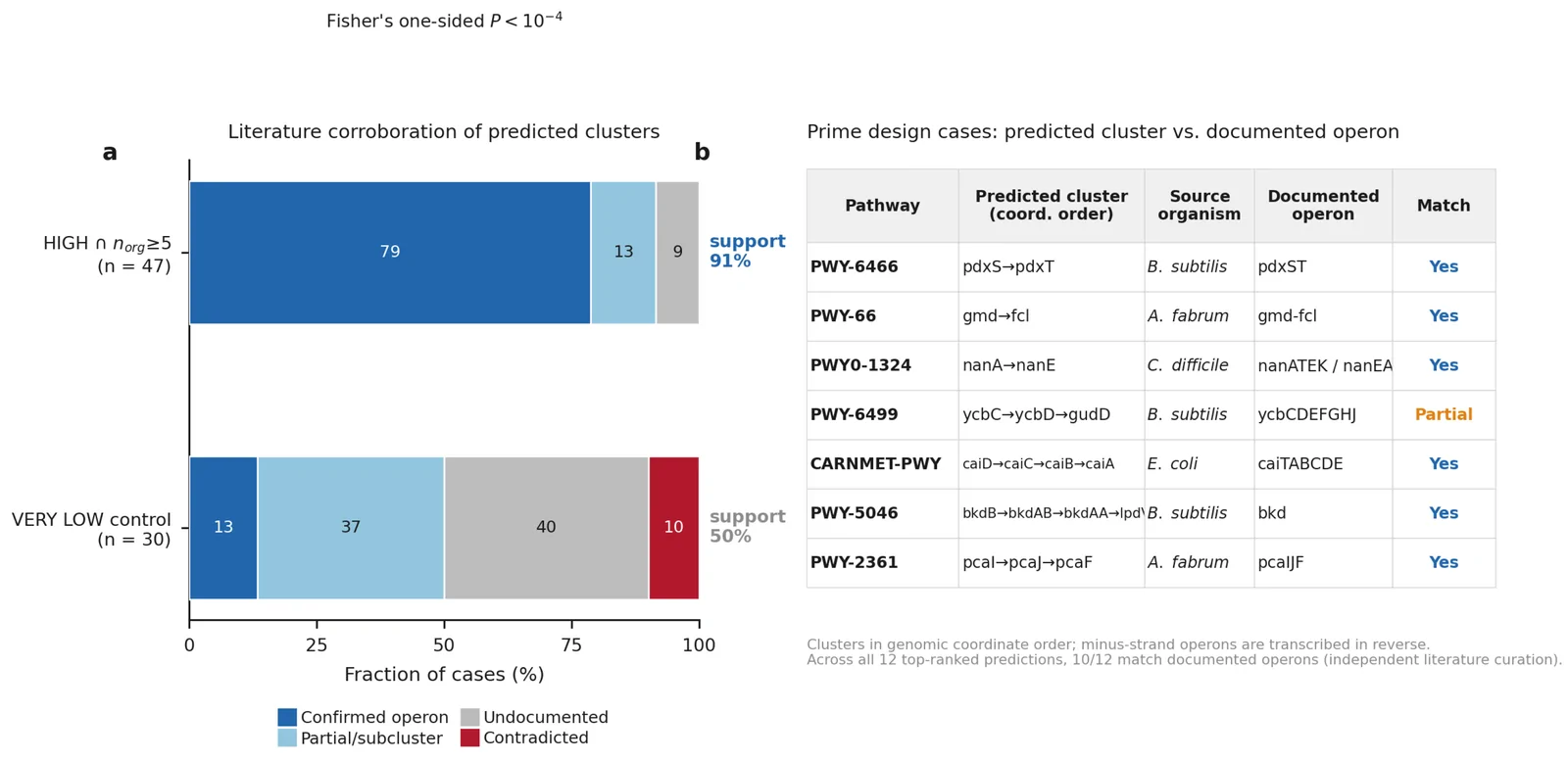
Agreement between high chromosomal clustering and independently documented bacterial operons or multi-gene clusters under a fixed labeling protocol. (**a**) Literature corroboration fractions for HIGH pathways shared by at least five organisms versus the expanded VERY LOW control; colors denote the fixed labels (confirmed operon, partial/subcluster, undocumented, contradicted), and the one-sided Fisher’s exact test *P* is shown above. (**b**) Prime design cases: predicted cluster in coordinate order versus the documented operon (source organism names in italic); “Match” indicates confirmed (Yes) or partial agreement.

High-ranking clusters include well-characterized transcriptional units: sialic acid utilization (*nan*) [26], branched-chain α-keto acid dehydrogenase (*bkd*) [27], pyridoxal 5′-phosphate synthesis (*pdxST*) [28], and GDP-L-fucose synthesis (*gmd*–*fcl*) [29] (Figure 3). Under the concordance protocol (HIGH with at least 5 organisms, n=47), thirty-seven were labeled confirmed and six partial (43/47; 91.5% support). In the expanded VERY_LOW control (n=30; see Section 2), 15 of 30 (50.0%) showed literature support (confirmed or partial), compared to 43/47 (91.5%) in HIGH pathways (one-sided Fisher exact p≈0.00006). Coordinate-based clustering therefore enriches for literature-supported operon-like units among high-scoring pathways; the comparison is protocol-defined and not a claim of unsupervised operon discovery.

### 3.4. Pathway-Table Order Is Not Chromosomal Synteny

**Figure 4:**
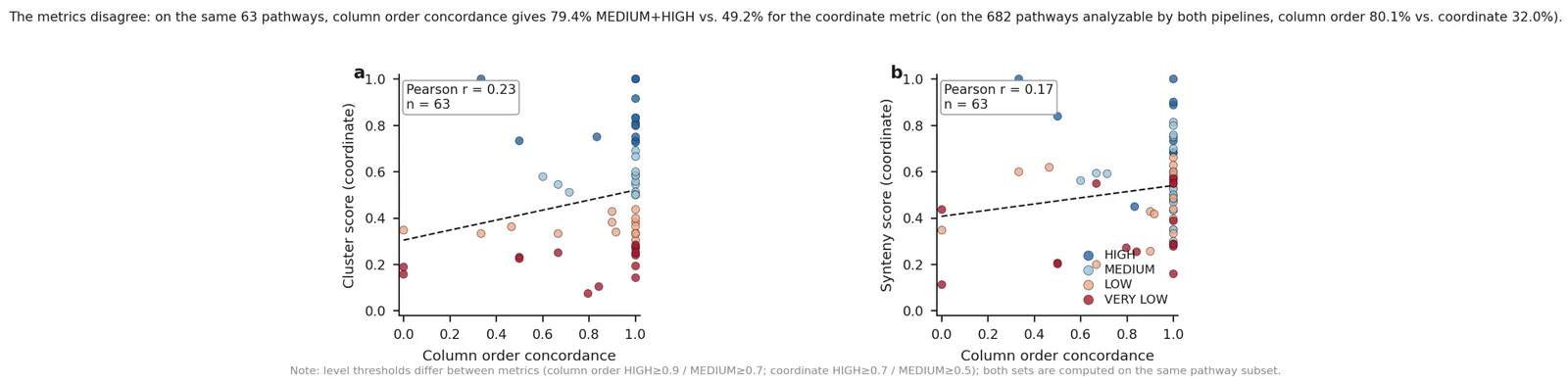
Column order concordance versus coordinate-based conservation scores on a paired pathway set. (**a**) Column order concordance vs. coordinate cluster score. (**b**) Column order concordance vs. coordinate synteny score. Points are colored by coordinate conservation level; dashed lines show linear fits with Pearson’s *r* and *n* annotated. Threshold differences are discussed in the text.

On the full 55-organism core panel, a direct re-run of the column-order metric yielded 682 analyzable pathways with 70.5% MEDIUM/HIGH under legacy thresholds (HIGH ≥ 0.9, MEDIUM ≥ 0.7) and 80.1% under thresholds aligned with the coordinate metric (HIGH ≥ 0.7, MEDIUM ≥ 0.5); on the same 682 pathways, coordinate cluster scores reached MEDIUM/HIGH in only 32.0%. The contrast is reproduced on the 45-organism species-deduplicated subset (72.4% vs. 29.7% on 569 pathways under the same legacy-threshold comparison) and on the legacy paired set (Figure 4). Applying the same numerical thresholds to both metrics on the paired set (n=63) yielded 90.5% (column order) versus 49.2% (coordinate), with a weak Pearson’s correlation r=0.23. Across all configurations, table order inflates apparent conservation and cannot substitute for coordinate clustering.

### 3.5. Design-Readiness Tiers as Layout Hypotheses

The tier counts on the core panel were T1 = 224, T2 = 83, T3 = 409, and T4 = 171 (Table 2). Pathways in T1 + T2 (307/887) retain substantial clustering evidence for module-oriented layout hypotheses; T3 pathways suggest multi-species module candidates; T4 pathways offer little native clustering guidance.

### 3.6. Construct Drafts for T1 Pathways

**Figure 5:**
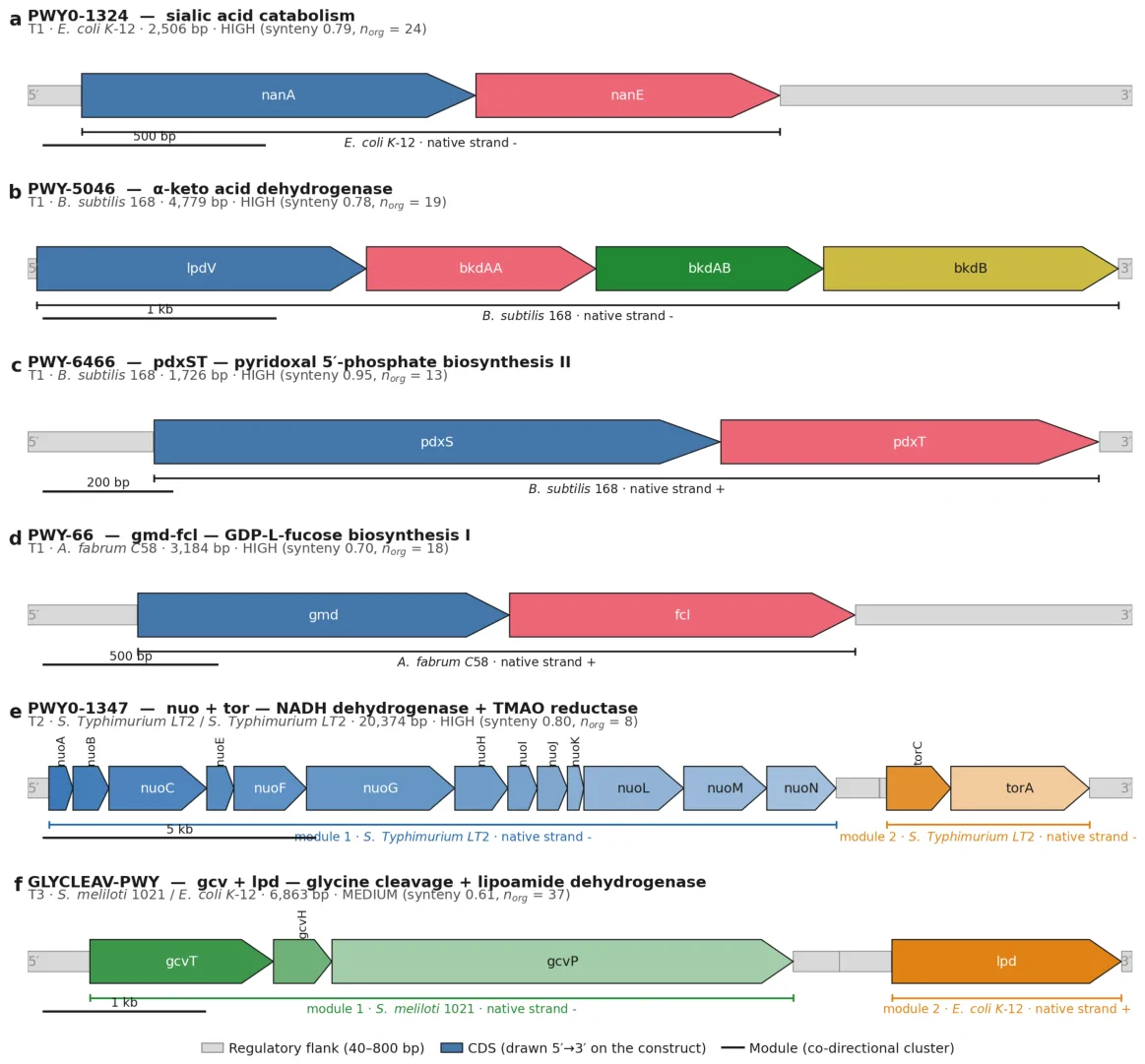
Illustrative construct drafts: recommended gene order, donor modules, and assembly overview. (**a**–**d**) T1 single-cluster drafts for documented operons (*nan*, *bkd*, *pdxST*, *gmd*–*fcl*); (**e**) a T2 draft combining two conserved modules; (**f**) a T3 draft combining modules from two donor species. Colored arrows are coding sequences drawn 5′ to 3′ on the construct; gray blocks are regulatory flanks (40–800 bp); brackets beneath the genes mark donor modules, annotated with the source organism (italic) and native strand.

For curated examples spanning T1–T3 (including *nan*, *bkd*, *pdxST*, and *gmd*–*fcl*), the draft procedure returned multi-kilobase records whose gene orders match published operon descriptions [26,27,28,29] (Figure 5). By applying the procedure to all 224 T1 pathways, sequence-backed drafts were obtained for 222 pathways. Two T1 pathways failed to yield multi-gene extracts under symbol-based matching. Drafts are starting points for experimental design, not validated portable units.

### 3.7. Operon TU Benchmark

To independently validate that SynPAL co-directional clusters correspond to real transcriptional units (TUs) rather than random spatial arrangements, we compared each cluster against BioCyc’s own TU annotations. Of 6065 (pathway, organism) clusters with at least two genes, 4906 (80.9%) had TU annotation available. Among these, 4632 (94.4%) had at least two cluster genes sharing a common TU identifier—meaning that in >94% of cases, SynPAL’s co-directional clustering recovered a set of genes that BioCyc independently curates as co-transcribed. This TU concordance rate holds across the taxonomic breadth of the core panel (e.g., 98% in *Bacillus subtilis*, 98% in *Mycobacterium tuberculosis*, 99% in *Lactococcus lactis*).

### 3.8. Assembly Feasibility

Of 222 sequence-backed T1 construct drafts, 221 (99.5%) passed all in silico assembly feasibility criteria (Table S5): total length ≤ 30 kb, GC content in [0.25, 0.75], primers with Tm in [40, 80] °C, and minimum Gibson overlap ≥ 15 bp. Construct lengths ranged from 2.3 kb to 31.4 kb (median 6.3 kb); a single draft (31.4 kb) exceeded the single-pot Gibson assembly range and was flagged for multi-round assembly. The mean GC content was 0.51, consistent with that of prokaryotic genomes. Each of the 222 drafts included at least one primer pair with Tm in the expected range.

### 3.9. Robustness on an Expanded Collection

On 9071 prokaryotic PGDBs, 1629 pathways were analyzable; 696 (42.7%) were classified as MEDIUM or HIGH. The tier counts were T1 = 869, T2 = 96, T3 = 452, and T4 = 212. The mean observed and null cluster scores were 0.492 and 0.309. The expanded survey preserves a selective, size-null-enriched landscape at greater taxonomic breadth.

### 3.10. Software Resource

**Figure 6:**
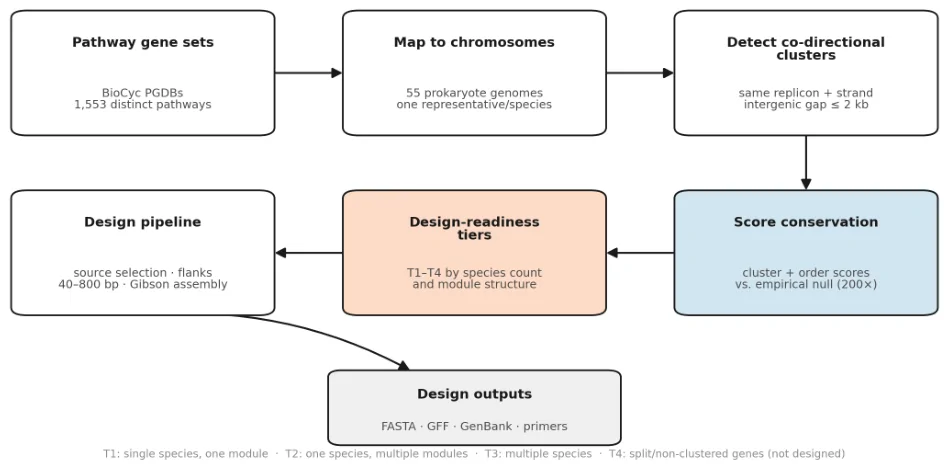
SynPAL workflow: pathway gene sets and chromosomal coordinates to cluster scores, layout tiers, and optional construct drafts. Arrows indicate the data flow between analysis steps; box shading distinguishes the statistical control step (blue) and the design-readiness tiering step (orange) from input/output steps (white/gray).

SynPAL is released as a compact Python toolkit for user GFF/GTF + FASTA and gene lists, with a scored core-panel database and a web-based tool to browse derived results (https://synpal.sjtu.bio, accessed on 28 August 2026). Figure 6 summarizes the workflow.

## 4. Discussion

Pathway CAD has advanced on the chemical axis—from rule-based retrosynthesis [1,2] to learning-based bioretrosynthesis [3] and integrated design portals that also touch genetic parts [4,5]. SynPAL addresses a narrower, complementary question: which co-directional chromosomal arrangements of a given gene list are recurrent enough to serve as layout priors? The empirical claim is deliberately modest. Conservation and literature concordance support using native neighborhoods as starting architectures; they do not demonstrate that a transferred arrangement is optimal or even functional in a new chassis.

Approximately one-third of core-panel pathways fall in the MEDIUM/HIGH range. That fraction is a selective signal, consistent with fluid bacterial chromosomes that nonetheless retain local functional neighborhoods [9,10]. Enrichment under a size-matched null, stability under FDR control, and protocol-defined recovery of classical operons argue against pure random co-occurrence of names. The column-order control shows that table presentation order—even under thresholds aligned to the coordinate metric—inflates MEDIUM/HIGH rates and correlates only weakly with coordinate scores.

Tiers T1–T4 compress clustering patterns into layout options for designers. Construct drafts close a practical loop from scores to editable sequence records. Polycistronic expression engineering shows that layout and coupling remain tunable after an initial architecture is chosen [19]; experimental optimization is outside SynPAL’s scope.

The limitations of this study must be made explicit. Symbol-based matching underestimates orthology and was not fully re-estimated with sequence orthologs here. This limitation is expected to affect taxa disproportionately: organisms with idiosyncratic or inconsistent gene naming conventions (notably some archaea, poorly annotated draft genomes, and taxa with extensive strain-specific locus-tag usage) will show artificially low cluster ratios, inflating the VERY_LOW tier and suppressing borderline MEDIUM scores. Taxon distribution analysis on the core panel shows a significant compositional difference between HIGH/MEDIUM and VERY_LOW pathways ($\chi^{2}=51.0$, p<0.0001), although the relative proportions of the domains Bacteria and Archaea do not differ significantly (p=0.11), indicating that the taxon bias operates within specific lineage groups rather than between domains. Symbol-matching failure rates confirm this: Bacteroidetes (55% of pathway–organism pairs with <50% gene recovery) and Chlamydiae (65%) show the lowest mapping rates, while Gammaproteobacteria (24% with <50% recovery) and Bacilli (41%) map well (Kruskal–Wallis p<0.0001). The expanded 9071-PGDB survey partially mitigates this by broadening taxonomic coverage, but a sequence-homology-based ortholog mapping using tools such as eggNOG-mapper [30] would be required for a fully unbiased estimate. The null model does not control regional density beyond gene-set size; the organism-level random draws reused across pathways of equal *k* introduce a non-independence that makes the FDR figure an empirical enrichment rate rather than a strictly independent test. A density-matched null sensitivity analysis confirms that the enrichment signal persists even when random gene sets are drawn from genomic windows of similar local density, consistent with the known influence of leading-strand bias and regional chromosomal architecture on bacterial gene arrangement [31]. Operon concordance used fixed rules on a HIGH-enrichment set and an expanded VERY_LOW control (*n* = 30, seed 42); support rates should not be read as discovery precision genome-wide. Primer suggestions are heuristic and do not account for salt, hairpin, or GC-content effects; re-derivation with Primer3 [25] is recommended before synthesis. No wet-lab titers are reported. Orthology-aware matching, denser spatial nulls, and coupling to reaction-CAD gene lists [3,5] are natural extensions.

## 5. Conclusions

Co-directional chromosomal clustering of metabolic pathway genes is a selective, non-random property of many—but not most—BioCyc pathways under a coordinate definition of neighborhood. SynPAL scores that property, maps it to layout-oriented tiers, recovers known operons under a stated protocol, and optionally produces construct drafts. These outputs are computational layout priors for synthetic construct design and complements to reaction-network pathway CAD, not experimental proofs of portable pathway units.

## Figures and Tables

**Table 1:** Pathway clustering levels on chromosomal coordinates.

Panel	Level	Count	%
Core (55 orgs, 887 pathways)	HIGH	89	10.0
	MEDIUM	201	22.7
	LOW	359	40.5
	VERY_LOW	238	26.8
	MEDIUM + HIGH	290	32.7
Resource scores (9071 orgs, 1629 pathways)	MEDIUM + HIGH	696	42.7

**Table 2:** Design-readiness tiers on the core panel.

Tier	Count	%	Meaning
T1	224	25.3	Full mapped set in one co-directional cluster in some organism
T2	83	9.4	Conserved partial modules (cluster_score ≥ 0.5)
T3	409	46.1	Weakly conserved clusters; heterologous cover candidates
T4	171	19.3	No 2-gene cluster; chemistry first

## Data Availability

Derived pathway scores, tiers, and analysis code for the core panel are available via https://github.com/lovivi/synpal (accessed on 28 August 2026; MIT License) and https://synpal.sjtu.bio (accessed on 28 August 2026). Pathway–genome associations originate from BioCyc [20,21]. Users may recompute scores from licensed BioCyc installs or from their own GFF/GTF, FASTA, and gene lists. Construct drafts contain extracted genomic intervals; redistribution follows the software release policy and does not include bulk PGDB flat file dumps.

## Supplementary Materials

The following supporting information can be downloaded at https://www.mdpi.com/article/10.3390/biotech15040075/s1, Table S1: full per-pathway scores for the 887-pathway core panel; Table S2: summary of the 222 T1 construct drafts; Table S3: gap-sensitivity analysis; Table S4: organism panel metadata; Table S5: per-construct in silico assembly feasibility metrics for the 222 T1 drafts; Table S6: taxon distribution of HIGH/MEDIUM versus VERY_LOW pathways with symbol-matching rates; Table S7: expanded operon validation control set (*n* = 30) with labels and evidence; Table S8: column-order versus coordinate conservation across the three pathway panels (apples-to-apples comparison).

## Abbreviations

The following abbreviations are used in this manuscript:

SynPAL	Synteny-Informed Pathway Assembly and Layout
PGDB	Pathway/genome database (BioCyc)
CAD	Computer-aided design
FDR	False discovery rate
T1–T4	Design-readiness tiers (layout hypotheses)
